# Toll-Like Receptor Activation in Immunity vs. Tolerance

**DOI:** 10.3389/fimmu.2015.00146

**Published:** 2015-04-02

**Authors:** Christophe M. Filippi

**Affiliations:** ^1^Immunology, Genomics Institute of the Novartis Research Foundation, San Diego, CA, USA

**Keywords:** toll-like receptors, immune stimulation, immunoregulation, autoimmune diseases, cancer immunotherapy, infection, probiotics, microbiome

After the discovery of Toll-like receptors (TLR) in the late 1990s, initial investigations were focused on understanding their role, stimulating immune responses against infectious agents. Yet, the human body is home to a myriad of TLR agonistic bacteria that have not only established symbiosis with the immune system but also likely contribute to the induction and maintenance of immune homeostasis by dampening immune responses. In fact, stimulation of TLRs might be critically involved in this process and ultimately contribute to preventing development of inflammatory and autoimmune diseases. Sander De Kivit and colleagues present an overview of those aspects, notably outlining the role of gut epithelial TLRs in the induction of immunity and the maintenance of tolerance (1). In addition, the authors highlight the mechanisms through which the gut microbiota regulates intestinal immune responses through interaction with TLRs. The capacity of probiotic microorganisms to modulate immunity via specific TLRs is further discussed in two articles by Julio Villena and Haruki Kitazawa. The authors explore the role of TLR interaction with immunobiotics for the regulation of intestinal inflammation in pigs, with a focus on TLR4 and *Lactobacillus jensenii* TL2937 (2). Interaction of probiotics with TLR3 to promote beneficial immunity beyond the gut in the respiratory tract is also examined (3). Specifically, inflammatory and immunoregulatory mechanisms conferred by *Lactobacillus rhamnosus* CRL1505 are presented along with their proposed effects increasing resistance to RSV infection while limiting immunopathology.

While TLRs stimulate and regulate immunity against infectious agents, they are also highly pursued targets for therapy of cancer due to their strong ability to activate multiple arms of the immune system, and in particular to stimulate those specific cellular and cytokine responses critical to anti-tumor immunity. However, the immune potentiating, anti-tumor effects of TLR agonists may be restrained by their parallel ability to trigger immune regulation. Dampening of immune responses may enable control of inflammatory damage or immunopathology, but the downside is a limiting effect on efficacy. The impact of this dichotomy on the use of TLR agonists for immunotherapy of cancer is discussed by Hailing Lu in a review article where key immune regulatory facets of TLR agonists are presented, which may impair their efficacy (4). The dual role of TLRs in cancer likely extends beyond the dichotomy between immune stimulation and regulation. Inflammatory processes triggered by TLR engagement may notably constitute a double-edged sword in elimination vs. development of tumors. In that regard, Erin Burns and Nabiha Yusuf contribute two opinion articles discussing TLR targeting for the treatment of cancer. The use of TLR agonists for skin cancer treatment is presented in the context of their impact on skin carcinogenesis, and the question of TLR tolerance is also discussed (5). Similarly, the dual effect of TLR agonists in breast cancer is examined, where TLR stimulation might beneficially activate the immune system but inflammatory processes may also promote tumor development, while TLR-conferred immune regulation may further curb anti-tumor immunity (6).

The mechanisms that underlie the immune regulatory properties of TLRs are not well understood. It is possible that the “default” response induced by TLR stimulation may vary between cell types, or depending on the microenvironment/anatomical location of TLR-expressing cells (as exemplified in gut mucosal tissue). Alexandra Zanin-Zhorov and colleagues described an immune regulatory mechanism conferred by TLR expression on T cells, which the authors review herein and discuss in the context of TLR-induced T cell effector functions (7). The role played by endogenous TLR ligands in conferring immune regulatory mechanisms is also presented. Nobuhiro Nakamoto and Takanori Kanai focus on a key organ, the liver, where various types of TLR-expressing cells are faced with continuous exposure to foreign antigens (8). The authors review immune stimulatory and regulatory effects of TLR signaling that coexist in the liver and influence liver health and disease. Elke Gülden and Li Wen concentrate on another organ, the pancreas, where TLR engagement also ultimately controls health and disease (9). Here, the beneficial and detrimental effects of TLR stimulation on type-1 diabetes are discussed, and notably the authors explore how endogenous TLR agonists can confer immune activation vs. regulation of autoreactive T cells. To close on the question of immune regulation conferred by TLRs, Himanshu Singh Chandel and colleagues contribute an opinion article investigating an alternative immune modulatory aspect that may influence the outcome of parasitic infection (10). The authors review mechanisms that underlie cross-talk between TLRs and CD40, and discuss how this interaction may determine the nature of anti-leishmanial immune responses and ultimately parasite elimination.

## Conflict of Interest Statement

The author declares that the research was conducted in the absence of any commercial or financial relationships that could be construed as a potential conflict of interest.
